# Melatonin and agomelatine alleviate ivermectin-induced mouse spermatogonia apoptosis via suppression of oxidative stress and calcium overload

**DOI:** 10.3389/fcell.2025.1713124

**Published:** 2026-01-07

**Authors:** Daniel Chavez Varias, Kennlee Orola, Soon-Jung Park, Sung-Hwan Moon, Seung Hee Shin, Buom-Yong Ryu

**Affiliations:** 1 Department of Animal Science and Technology, Chung-Ang University, Anseong-Si, Republic of Korea; 2 Biosolvix, Seoul, Republic of Korea

**Keywords:** ivermectin toxicity, melatonin analogs, oxidative stress, mitochondrial dysfunction, male germ cell

## Abstract

**Introduction:**

Drug toxicity poses a significant threat to male fertility, and its mechanism is often associated with redox imbalance and mitochondrial dysfunction. Ivermectin (IVM), an anthelmintic increasingly explored for new therapeutic applications, induces apoptosis and impairs proliferation in spermatogonia via mitochondria-associated cellular injury at high concentrations in vitro. This study evaluated the protective effects of melatonin, agomelatine, and pinoline, as mitochondria-directed cytoprotectants.

**Methods:**

Cultured type B spermatogonia were pretreated with 1 μM melatonin, agomelatine, or pinoline for 24 h under low-serum conditions, followed by exposure to 16 μM IVM. Cell proliferation was assessed by cell counting and Ki67 immunocytochemistry. Mechanistic analyses included fluorescence imaging of reactive oxygen species (ROS) using 2',7'-dichlorodihydrofluorescein diacetate, cytosolic Ca^2+^ using Fluo-4, AM, and mitochondrial membrane potential (ΔΨm) using tetramethyl rhodamine ethyl ester. Mitochondrial function was evaluated using Seahorse assays, and apoptosis was evaluated by caspase cleavage, the BAX/BCL-2 ratio, and Cytochrome c levels by Western blotting.

**Results:**

Unlike pinoline, melatonin and agomelatine effectively suppressed IVM-induced oxidative stress and Ca^2+^ overload, while restoring mitochondrial membrane potential (ΔΨm), mitochondrial mass, and oxidative phosphorylation. These protective effects led to reduced apoptosis and enhanced cell proliferation. Structural differences among the three compounds indicate that the methoxy group and N-acetyl side chain are critical determinants of mitochondrial protection under redox stress.

**Conclusion:**

Melatonin and agomelatine protect the male reproductive system from drug-induced toxicity by restoring redox homeostasis and mitochondrial function. These findings provide mechanistic insight into melatonin-based therapeutic strategies and the development of fertility preserving agents targeting mitochondria-mediated cellular injury.

## 1 Introduction

Pharmacological drugs can impact spermatogenesis, the complex process of functional gamete development in the testis, at toxic doses. They may affect male fertility via pre-testicular (i.e., endocrine disruption), testicular (i.e., specific toxicological mechanisms in any cell within the testicular niche microenvironment), and post-testicular (i.e., sperm injuries during transport and secretion) mechanisms (Sousa et al., 2017). Redox imbalances usually mediate testicular toxicity mechanisms in spermatogonial stem cells and progenitor spermatogonia, which must constantly divide and differentiate to perpetuate spermatogenesis (Evans et al., 2021).

**FIGURE 1:**
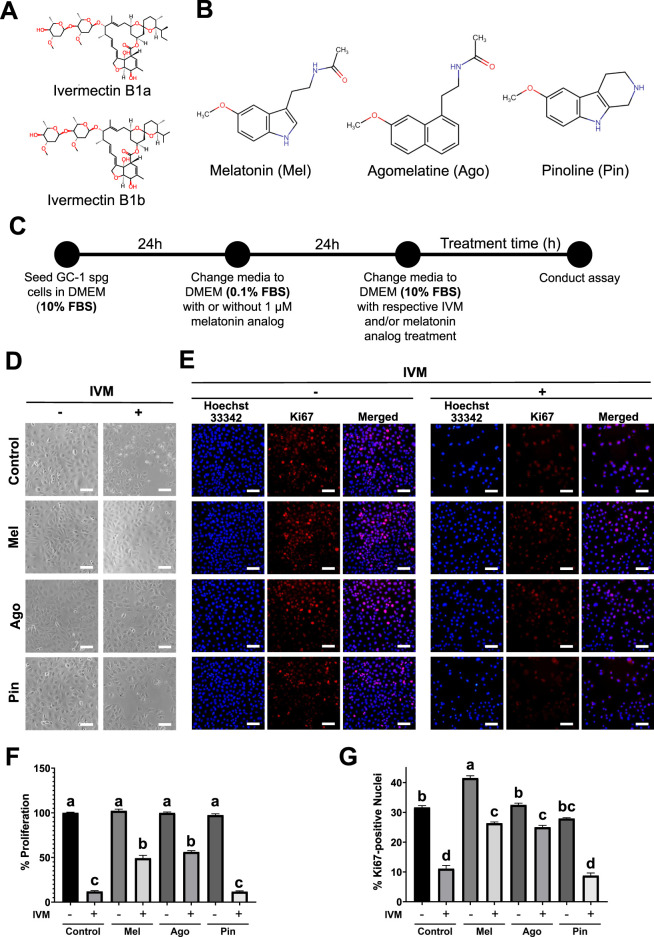
Effects of melatonin and agomelatine on proliferation loss in GC-1 spermatogonia (spg) treated with ivermectin (IVM) for 24 h **(A)** Chemical structure of IVM isomers. **(B)** Chemical structures of melatonin and its analogs, agomelatine and pinoline. **(C)** Schematic overview of the cell culture and experimental design. **(D)** Brightfield microscopy images showing cytoplasmic morphology. **(E)** Immunofluorescence images of Ki67 nuclear translocation. **(F)** Quantification of relative cell proliferation (n = 3). **(G)** Quantification of Ki67-positive nuclei (n = 3) in GC-1 spg treated with either control, melatonin, agomelatine, or pinoline with or without 16 μM IVM. IVM treatment conditions are denoted as positive (+) or negative (−). Scale bars: 100 µm. Data are presented as means ± SEM. Significant differences are indicated by different letters **(a–d)** at *p* < 0.05.

Ivermectin (IVM, Figure 1A), a broad-spectrum antiparasitic for humans and other animals, is gaining recognition as a repurposed drug against lumpy skin disease in cattle, malaria, and several types of cancer (Greenhalgh et al., 2025). To maximize IVM’s potential beyond its anthelmintic activity, studies must demonstrate strategies to widen its margin of safety. This will ensure the absence of toxicity even if the determined effective therapeutic dose exceeds the current approved dose, i.e., 200 μg/kg (Caly et al., 2020; Schmith et al., 2020).

We previously elucidated IVM’s novel and rapidly occurring mechanism of toxicity involving endoplasmic reticulum (ER) stress in mouse spermatogonia, which is inhibited by selenium (Se) (Chavez Varias et al., 2024). However, Se-based therapeutics with immediate bench-to-bedside potential remain limited; thus, discovering alternative protective approaches is still highly relevant (Zhang et al., 2026). Based on the previously detailed mechanism—where the mitochondrion is affected downstream of the Ip3r1–Grp75–Vdac1 axis—mitochondrial function emerges as a promising alternative target. To explore this, we investigated strategies aimed at enhancing mitochondrial resilience to prevent the unprecedented decline in mouse spermatogonia proliferation *in vitro*. This led us to study melatonin’s effectiveness as an antioxidant and its ability to access the mitochondria. Accordingly, we examined whether melatonin cotreatment could mitigate IVM-induced toxicity. Evaluating drug toxicity via antioxidant cotreatment provides a straightforward, mechanistically informative approach to define oxidative stress–related testicular impairment (Esmaeli et al., 2025).

The pineal gland naturally synthesizes melatonin, the “sleep hormone” responsible for regulating mammalian circadian rhythm (Luo et al., 2022). However, its therapeutic potential extends to ameliorating metabolic and neurodegenerative diseases by modulating mitochondrial function and inhibiting free radical accumulation (Jimenez-Delgado et al., 2021; Salagre et al., 2025). Multiple cytochemical studies support melatonin’s ability to access mitochondria more effectively than other antioxidants (Verma et al., 2023; Xiong et al., 2023), making it more inclined to prevent downstream mitochondria-associated cellular injuries (Supinski et al., 2020; Reiter et al., 2024; Yang et al., 2025). Specifically, melatonin preferentially mitigates mitochondrial permeability transition pore (mPTP) opening, mitochondrial Ca^2+^ overload, mitochondrial reactive oxygen species (ROS) accumulation, electron transport chain (ETC.) malfunction, and consequences of large-scale mitochondrial DNA deletion (Lei et al., 2024; Reiter et al., 2024). But in pharmacological toxicity, only a few studies have demonstrated melatonin as a mitochondria-targeted cytoprotectant in detail (Xing et al., 2021; Arinno et al., 2023; Cheng et al., 2024).

Some melatonin analogs arise from modifying the molecular structure of melatonin to increase its therapeutic value by achieving certain effects, such as prolonged half-life (Luo et al., 2022). Other analogs are possible, such as naturally occurring melatonin metabolites (Jiang et al., 2009; De La Fuente Revenga et al., 2015). Agomelatine and pinoline are analogs and are contrasting in chemical structure and signal transduction mechanisms (Table 1; Figure 1B). This merits the investigation regarding whether these differences influence the ability of the analogs to target the mitochondria in mitigating IVM toxicity in mouse male germ cells.

**TABLE 1:** Comparative structural and functional features of melatonin, agomelatine, and pinoline.

Structural feature	Melatonin	Agomelatine	Pinoline	References
A. Structural features				
Methoxy group	Present	Present	Present	Hoashi et al. (2021)
Indole core	Present	Absent	Present	Hoashi et al. (2021)
Naphthalene bioisostere core	Absent	Present	Absent	​
N-acetyl side chain	Present	Present	Absent	Hoashi et al. (2021)
Secondary amine	Absent	Absent	Present	De La Fuente Revenga et al. (2015)

Functional feature	Melatonin	Agomelatine	Pinoline	References
B. Functional features				
MT1/MT2 receptor agonist activity	Yes	Yes	No	Gao et al. (2023), Park et al. (2025)
5-HT receptor antagonist activity	No	Yes	No	Gao et al. (2023), Sun et al. (2025)
5-HT reuptake inhibition	No	No	Yes	​

In this study, we assessed melatonin and its analogs, agomelatine and pinoline, in ameliorating IVM-induced loss of proliferative ability and cell death in mouse spermatogonia. We investigated the action of small molecules on ROS accumulation, Ca^2+^ overload, mitochondrial membrane potential, and mitochondrial bioenergetics. The analogs that restored mitochondrial function and improved cell proliferation enabled us to present new insights on pharmacological approaches in alleviating IVM toxicity related to male fertility.

## 2 Materials and methods

### 2.1 Cell culture

Mouse type B GC-1 spermatogonial (spg) cells (CRL-2053, American Type Culture Collection, Manassas, VA, USA) were cultured in complete media using Dulbecco’s modified Eagle’s medium (DMEM, L0103-500; Biowest, Nuaillé, France) with 10% fetal bovine serum (FBS, S1480; Biowest) and penicillin/streptomycin (15140122, Gibco, Waltham, MA, USA) in 5% CO_2_ at 37 °C. For experimental assays, cells were seeded at 20 000 cells/mL in phenol-free DMEM (LM001-10; Welgene, Gyeongsan-si, Korea). After 24 h, the medium was replaced: cells were washed with Dulbecco’s phosphate-buffered saline (DBPS, 14080055; Life Technologies, Grand Island, NY, USA) and incubated in quiescing media (phenol-free DMEM with 0.1% FBS with penicillin/streptomycin). Cells were then pretreated with or without 1 μM melatonin (D5250; Sigma-Aldrich), agomelatine (GC17981; GlpBio, Montclair, CA, USA), or pinoline (N1040-01; Natural Product Institute of Science and Technology, Korea) in quiescing media for 24 h. Subsequently, cells were treated with complete media containing either 0 μM or 16 μM ivermectin (IVM, 18,898; Sigma-Aldrich), with or without 1 μM melatonin or analogs, for durations specified in each experiment. The 16 μM IVM dose was determined based on our previous work (Figure 1C). No human participants nor live animals were involved at any point in this study.

### 2.2 Measuring GC-1 spg proliferation via trypan blue exclusion assay (TBEA)

TBEA was performed to assess GC-1 spg proliferation after 1 cell cycle (24 h) while being exposed to IVM, with or without co-treatment of melatonin or its analogs. After 24 h of drug exposure, cells were harvested using trypsin-EDTA (25200072; Invitrogen, Carlsbad, CA, USA), centrifuged, and suspended in cold PBS. Single-cell suspensions (10 μL) were mixed with trypan blue (10 μL) (15–250–061; Gibco) and loaded into a hematocytometer. Viable cells (unstained) were manually counted under a light microscope, and proliferation (%) relative to the control was calculated using Equations 1, 2:

$$\%\text{ Proliferation}=\frac{\text{Number}\;\text{of}\;\text{Harvested}\;\text{cells}}{\text{Number}\;\text{of}\;\text{Seeded}\;\text{cells}}\times 100$$ (1)

$$\text{Relative }\%\text{ proliferation}=\frac{{\text{Proliferation}}_{\text{Experimental}\;\text{group}}}{{\text{Proliferation}}_{\text{Control}\;\text{group}}}\times 100$$ (2)

### 2.3 Immunocytochemistry (ICC) analysis of proliferation marker

After 24 h of drug treatment, GC-1 spg cultured in 12-well plates were fixed in 4% paraformaldehyde, washed three times with DPBS, and overlaid with a 1 mm-thick layer of 100 mM sucrose in 1× DPBS before drying on a hot plate surface at 60 °C. Cells were permeabilized with 0.1% Triton X-100 with 5% bovine serum albumin in DPBS (v/v) for 10 min at room temperature (RT, 23 °C–25 °C) before blocking with 5% bovine serum albumin in 1× DPBS (w/v) for 1 h. Proliferating cells were stained with anti-Ki67 primary antibody (ab66152; abcam, Cambridge, UK, RRID: AB_1141192) for a minimum of 8 h at 4 °C, then washed thrice in 5-min intervals using 5% bovine serum albumin in 1× DPBS (w/v). Alexa Fluor 568-conjugated secondary antibody (1:1,000, A11011; Invitrogen, RRID: AB_143157) diluted in 5% bovine serum albumin in 1× DPBS (w/v) was applied for at least 4 h at 4 °C. Nuclei were counterstained with Hoechst 33,342 (B2261; Sigma-Aldrich) during the first two of three washes. Fluorescent images were captured using a TE2000-U fluorescence microscope (Nikon, Tokyo, Japan), and Ki67-positive nuclei were manually counted using ImageJ software (National Institutes of Health, Bethesda, MD, USA) with red-blue thresholding. Data were normalized to the total nuclei count using Equation 3:

$$\%\text{ Ki}67‐\text{positive nuclei}=\frac{\text{Ki}67‐\text{positive}\;\text{nuclei}}{\text{Total}\;\text{nuclei}}\times 100$$ (3)

### 2.4 Intracellular ROS detection

After 3 h of drug treatment, the fluorescent probe dichlorodihydrofluorescein diacetate (DCFDA, D6883; Sigma-Aldrich) was used to visualize ROS accumulation. GC-1 spg were washed twice with DPBS before incubation at 37 °C in 10 μM DCFDA dissolved in phenol-free DMEM and 1% FBS. Hoechst 33,342 with a final concentration of 2 μg/mL was mixed with the DCFDA media before imaging. Cold DPBS was used as a final wash.

### 2.5 Cytosolic Ca^2+^ imaging

Ca^2+^ within the cytosol was imaged using Fluo-4, AM (F14201; Sigma-Aldrich), after 3 h of drug exposure. Residual media were washed twice using phenol-free DMEM, Ca^2+^, and Mg^2+^. The same medium was used to dissolve 1 μM Fluo-4, AM, which was incubated with the cells for 1 h at 37 °C. After loading the probe, three washing steps were conducted with phenol-free DMEM. The cells were incubated for 30 min using the same media. The fluorescent cells were imaged immediately after washing.

### 2.6 Inner mitochondrial membrane potential (ΔΨm) analysis

Tetramethylrhodamine methyl ester (TMRE; T669, Thermo Fisher Scientific) was used to visualize ΔΨm of GC-1 spg after drug exposure for 3 h. The cells were incubated in TMRE (1 μM), MitoTracker (200 nM) (M7514; Invitrogen), and Hoechst 33,342 (2 μg/mL) in phenol-free DMEM. Excess probes were washed once with DPBS before imaging.

### 2.7 Semi-quantitation of live cell fluorescence microscopy images

The percentage area of the fluorescence of images from the ROS, Ca^2+^, and ΔΨm assays was semi-quantified with ImageJ (Chavez Varias et al., 2024). Fluorescence images captured with a fluorescence microscope were converted to 16-bit images, then to 8-bit binary images. At least three random fields were analyzed. Cell numbers per field were recorded manually. Equation 4 was used to normalize the area of fluorescence in DCFDA-, Fluo-4, AM-, MitoTracker- and TMRE-stained cells.

$$\%\text{ Area of fluorescence per }100\text{ cells}=\frac{\%\;\text{Area}}{\text{Total}\;\text{number}\;\text{of}\;\text{cells}\;\text{in}\;\text{the}\;\text{field}}\times 100$$ (4)

Equation 5 was modified from the previous formula to account for ΔΨm (TMRE) relative to mitochondrial mass:

$$\%\;\Delta \Psi m\text{ to Mitochondrial Mass Ratio}=\frac{\%\;{\text{Area}}_{\text{TMRE}}}{\%\;{\text{Area}}_{\text{MitoTracker}\;}}\times 100$$ (5)

### 2.8 Seahorse assay

The mitochondrial function of GC-1 spg was measured using an Agilent Seahorse XF Cell Mito Stress test kit (103,792–100; Agilent, Santa Clara, CA, USA) after 3 h of drug treatment. First, Seahorse XFe96 cell culture microplate surfaces were coated with 0.1% (w/v) gelatin before seeding with 7,000 GC-1 spg cells per well. Following the treatment, the oxygen consumption rate (OCR) was measured with a Seahorse Extracellular Flux Analyzer (Agilent). Briefly, the cells were incubated in assay medium at 37 °C without CO_2_ for 1 h. Oligomycin (final concentration: 0.5 μM), carbonyl cyanide-p-trifluoromethoxyphenylhydrazone (FCCP; final concentration: 1 μM), and rotenone/antimycin A (R/AA; final concentration: 0.5 μM) were loaded in respective ports and were injected into the wells. OCR was measured at the standard timepoints. After measurement, the values were normalized using the sulforhodamine B (SRB) assay. Essentially, 10% (v/v) trichloroacetic acid-fixed cells were stained with 1.4% (w/v) SRB in 1% (v/v) acetic acid, followed by dye solubilization in 10 mM Tris buffer, pH 10.5. Absorbance at 690 nm was measured using a SpectraMax 190 microplate spectrophotometer (Molecular Devices, San Jose, CA, USA).

### 2.9 Western blotting

After 3 h of treatment, cells were scraped, centrifuged for 6 min at 600 × *g*, and resuspended in cold DPBS before a second centrifugation for 1 min at 19,000 × *g*. Cell lysis was performed using total extraction buffer containing dithiothreitol, phenylmethylsulfonyl fluoride, and a protease inhibitor cocktail (1862206; Thermo Fisher Scientific). The lysates underwent vigorous pipetting, flash-freezing in liquid nitrogen, and thawing at RT, followed by centrifugation at 19,000 *g* for 30 min at 4 °C. Protein concentrations in the supernatant were measured using the Bradford assay (5000006; Bio-Rad, Hercules, CA, USA). Equal amounts of protein were loaded and electrophoresed gradient sodium dodecyl sulfate-polyacrylamide gel (6, 12, 15%; 3:4:3) alongside a protein ladder (SM306; BioFACT, Daejeon, Korea) and transferred to Immobilon®-P methanol-activated polyvinylidene difluoride membranes (IPVH00010; Millipore, Billerica, MA, USA), which were previously activated in methanol (322,415; Sigma-Aldrich) for 1 min. Membranes were blocked with 5% skim milk in DPBS, washed with PBS-T (DPBS containing 0.2% Tween 20) for 30 min, and incubated with primary antibodies. After further PBS-T washes, membranes were incubated with appropriate secondary antibodies for 2 h at RT, membranes were washed three times again and treated with WestGlow™ Femto solution (BWF0100; Biomax, Guri-si, Korea) before imaging with e-Blot Touch Imager (e-Blot Life Science, Shanghai, China). The following antibodies were used: anti-caspase-9 (9504; Cell Signaling Technology [CST], RRID: AB_227591), anti-caspase-8 (D35G2) (4790; CST, RRID: AB_10545768), anti-caspase-7 (9492; CST, RRID: AB_2228313), anti-caspase-3 (D3R6Y) (14,220; CST, RRID: AB_2798429), anti-BCL-2 (D17C4) (3498; CST, RRID: AB_2290566), anti-BAX (D3R2M) (14,796; CST, RRID: AB_2721185), anti-Cytochrome c (ab90529; abcam, RRID: AB_10673869), anti-α-tubulin [DM1A] (ab7291; abcam, RRID: AB_2241126), anti-rabbit-HRP conjugated (7074, CST, RRID: AB_2099233), and anti-mouse-HRP conjugated (7076; CST, RRID: AB_330924).

### 2.10 Statistical analysis

GraphPad Prism v9.5.1 (GraphPad, La Jolla, CA, USA) was used for statistical analyses. At least three biological replicates were carried out in every experiment. Data are shown as mean ± standard error of the mean (SEM). Except for Seahorse assay where we employed two-way analysis of variance, one-way analysis of variance was applied to determine statistical significance at 5%. Multiple comparisons between groups were performed using Tukey’s Honest Significant Difference. Groups were assigned different letters to indicate statistical significance (p < 0.05).

## 3 Results

### 3.1 Melatonin and agomelatine promote the proliferation of GC-1 spg subjected to cytotoxic IVM concentrations

In a previous study, we determined 16 μM as the concentration of IVM that inhibits proliferation in mouse type B spermatogonia (Chavez Varias et al., 2024). To evaluate whether melatonin or its analogs could counteract this effect, GC-1 spg were co-treated with 16 μM IVM and 1 μM of melatonin, agomelatine, or pinoline. After 24 h−approximately 1 cell cycle−melatonin and agomelatine, but not pinoline, markedly restored GC-1 spg proliferation compared to IVM-only treated cells. Treatment with 1 μM melatonin or its analogs alone did not substantially affect proliferation relative to the control. Cytoplasmic vacuolization observed after 24 h of IVM exposure was noticeably reduced by co-treatment with melatonin or agomelatine. (Figures 1D,F). In addition, the nuclear translocation of Ki67, a marker of cell proliferation suppressed by IVM, was restored to near control levels in cells co-treated with melatonin or agomelatine. Melatonin alone increased the proportion of Ki67-positive nuclei compared to control, whereas pinoline alone caused a slight but marked decrease (Figures 1E,G). However, total cell proliferation (as determined using TBEA) did not differ substantially among these groups (Figure 1F).

### 3.2 Melatonin analogs inhibit ROS generation to different extents in GC-1 spg exposed to cytotoxic concentrations of IVM

**FIGURE 2:**
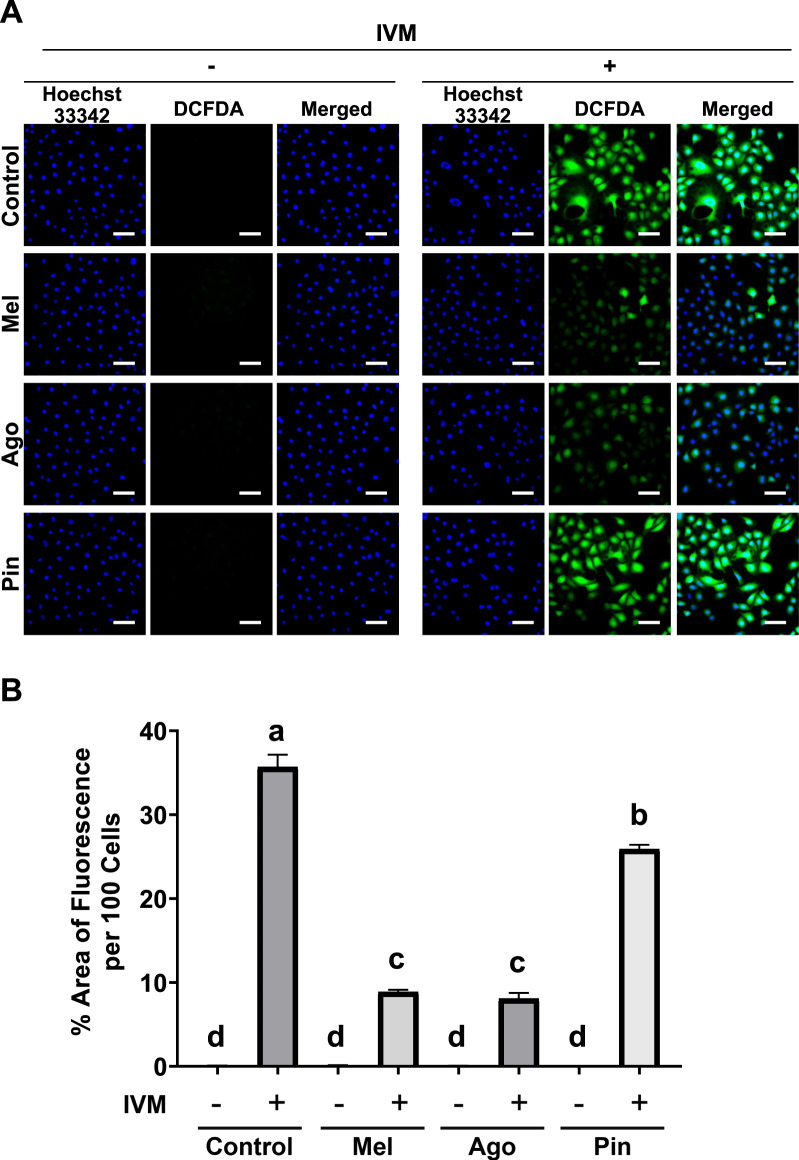
Inhibition of intracellular reactive oxygen species (ROS) generation by melatonin and agomelatine in GC-1 spermatogonia (spg) treated with ivermectin (IVM) for 3 h **(A)** Fluorescence microscopy images of GC-1 spg labeled with DCFDA. **(B)** Semi-quantification of DCFDA fluorescence intensity in GC-1 spg treated with either control, melatonin, agomelatine, or pinoline with or without 16 μM IVM (n = 5). IVM treatment conditions are denoted as positive (+) or negative (−). Scale bars: 100 µm. Data are shown as means ± SEM. Significant differences are indicated by different letters **(a–d)** at *p* < 0.05. Mel, melatonin; Ago, agomelatine; Pin, pinoline.

IVM-induced male germ cell toxicity is strongly associated with rapid and severe redox imbalance. To assess the ability of melatonin and its analogs to inhibit intracellular ROS accumulation in GC-1 spg, fluorescence microscopy was performed using DCFDA as a probe. Groups not exposed to IVM, including those treated with melatonin or its analogs alone, exhibited negligible fluorescence. In IVM-treated groups, both melatonin and agomelatine substantially reduced fluorescence intensity by more than threefold compared to cells treated with the IVM-only group. In contrast, co-treatment with pinoline resulted in a modest but statistically significant reduction in fluorescence compared to IVM alone (Figures 2A,B).

### 3.3 Melatonin and agomelatine inhibit IVM-induced intracellular Ca^2+^ accumulation

**FIGURE 3:**
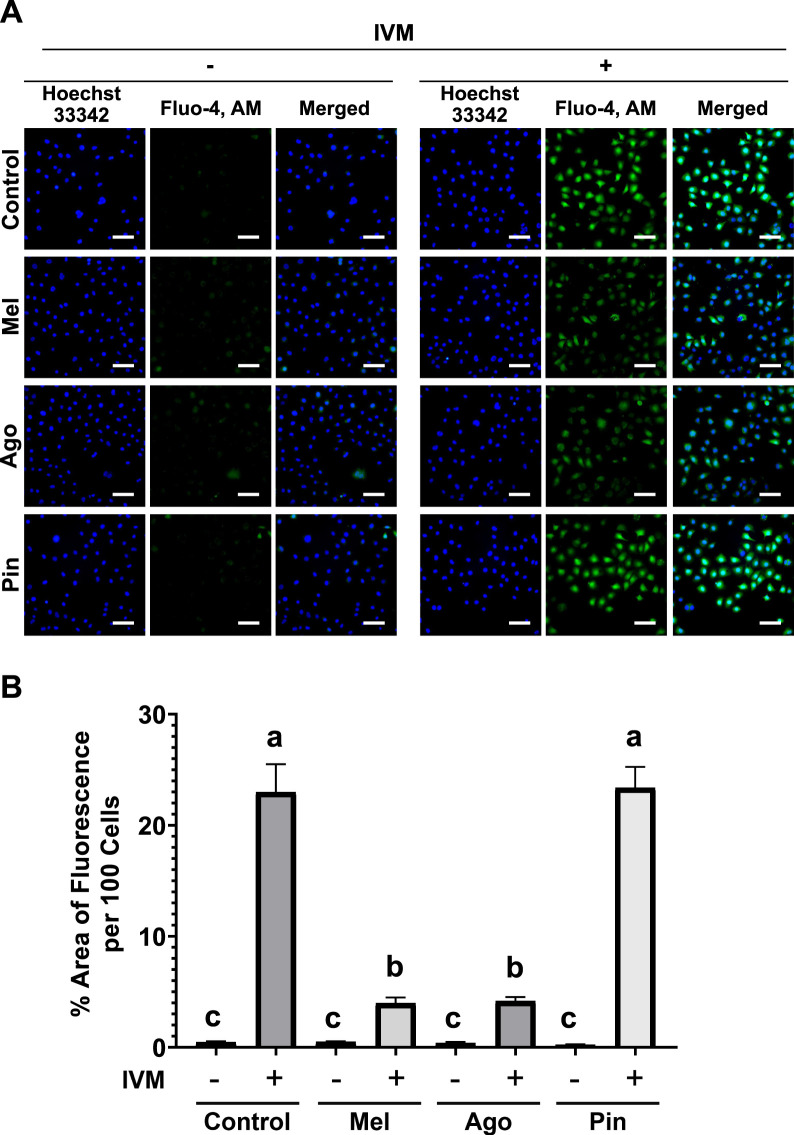
Melatonin and agomelatine inhibit cytoplasmic Ca^2+^ accumulation in GC-1 spermatogonia (spg) treated with ivermectin (IVM) for 3 h **(A)** Fluorescence microscopy images of GC-1 spg cells labeled with Fluo-4, AM. **(B)** Semi-quantification of Fluo-4, AM fluorescence intensity in GC-1 spg treated with either control, melatonin, agomelatine, or pinoline with or without 16 μM IVM (n = 5). IVM treatment conditions are denoted as positive (+) or negative (−). Scale bars: 100 µm. Data are presented as means ± SEM. Significant differences are denoted by different letters **(a–c)** at *p* < 0.05. Mel, melatonin; Ago, agomelatine; Pin, pinoline.

To assess intracellular Ca^2+^ levels, an important mediator of IVM-induced apoptosis in male germ cells, Fluo-4, AM was used to monitor calcium signals in GC-1 spg treated with IVM, with or without melatonin analogs. Minimal fluorescence was observed in cells treated with melatonin, agomelatine, or pinoline alone, all of which were comparable to the untreated control. In contrast, cells co-treated with melatonin or agomelatine and IVM exhibited nearly a six-fold reduction in fluorescence intensity compared to those treated with IVM alone. However, cells exposed to both pinoline and IVM showed no reduction in fluorescence intensity relative to the IVM-only group (Figures 3A,B).

### 3.4 Melatonin and agomelatine restore ΔΨm and mitochondrial mass in IVM-treated GC-1 spg

**FIGURE 4:**
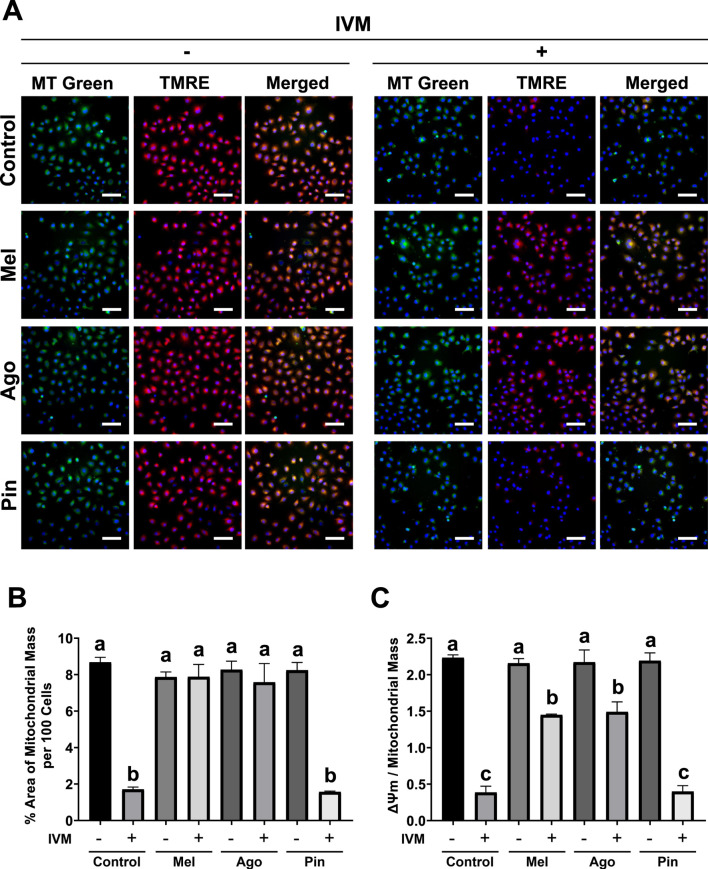
Melatonin’s and agomelatine’s recovery of ΔΨm and mitochondrial mass in GC-1 spermatogonia (spg) treated with 16 μM ivermectin (IVM) for 3 h **(A)** Fluorescence microscopy images of GC-1 spg stained with tetramethylrhodamine ethyl ester (TMRE) and/or MitoTracker and Hoechst 33,342. **(B)** Semi-quantification of MitoTracker fluorescence intensity (n = 5), and **(C)** ratio of TMRE to MitoTracker fluorescence intensity in GC-1 spg treated with either control, melatonin, agomelatine, or pinoline with or without 16 μM IVM (n = 5). Scale bars: 100 µm. IVM treatment conditions are denoted as positive (+) or negative (−). Data are shown as means ± SEM. Significant differences are denoted by different letters **(a–c)** at *p* < 0.05. Mel, melatonin; Ago, agomelatine; Pin, pinoline

Mitochondrial health parameters in GC-1 spg, specifically membrane potential (ΔΨm) and mitochondrial mass, were assessed by co-staining with TMRE (red) and MitoTracker (green), respectively. At cytotoxic concentrations of IVM, both ΔΨm (normalized to mitochondrial mass) and mitochondrial mass were substantially reduced. However, in cells co-treated with melatonin or agomelatine, mitochondrial mass remained unaffected, and the reduction in ΔΨm was considerably less than that observed in the IVM-only group. In contrast, co-treatment with pinoline did not substantially improve either parameter compared to IVM treatment alone (Figures 4A–C).

### 3.5 Melatonin and its analogs restore oxidative phosphorylation to varying degrees in IVM-treated GC-1 spg

**FIGURE 5:**
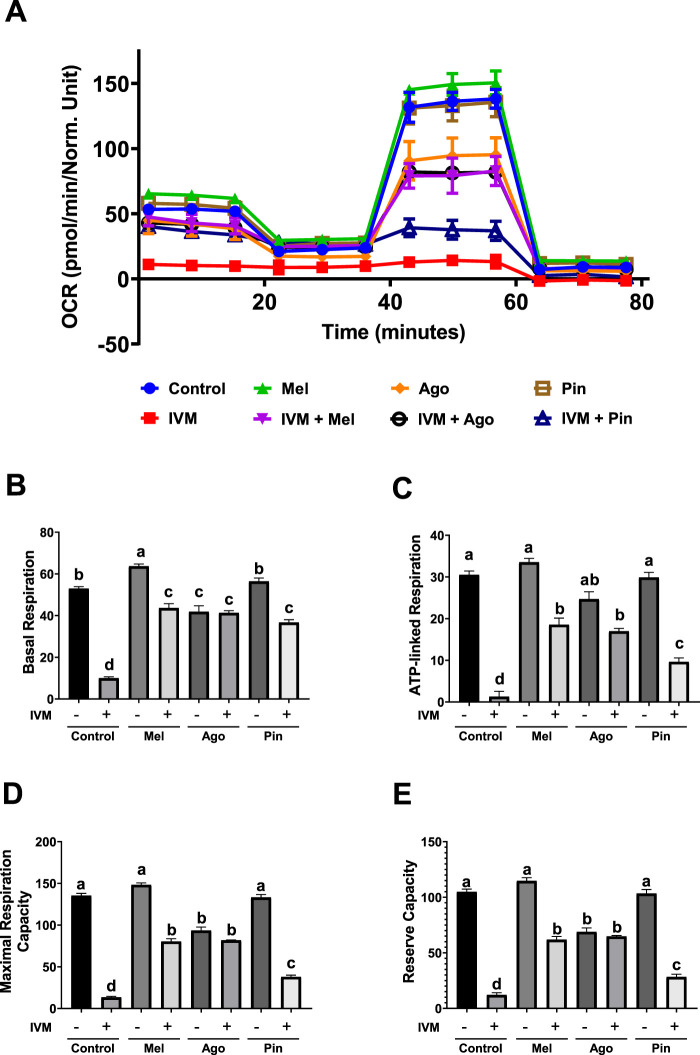
Seahorse assay of GC-1 spermatogonia (spg) treated with or without melatonin analogs and/or ivermectin (IVM) for 3 h **(A)** Oxygen consumption rate (OCR) plot following sequential injections of oligomycin, FCCP (carbonyl cyanide-p-trifluoromethoxyphenylhydrazone), and rotenone/antimycin A (R/AA). **(B)** Basal respiration, **(C)** ATP-linked respiration, **(D)** maximal respiration capacity, and **(E)** reserve capacity calculated from OCR data (n = 3). Data are presented as means ± SEM. Significant differences are indicated by different letters **(a–d)** at *p* < 0.05. Mel, melatonin; Ago, agomelatine; Pin, pinoline.

OCRs were measured using the Seahorse assay to evaluate mitochondrial respiration in GC-1 spg exposed to toxic concentrations of IVM with or without melatonin or its analogs. The basal respiration of the IVM-only group, as indicated by OCR during the first 20 min, was nearly zero compared to other groups. The melatonin-only group exhibited substantially higher basal respiration than the control, whereas the pinoline-only group had OCR levels comparable to the control. In contrast, the agomelatine-only group demonstrated noticeably lower basal respiration than the control. The agomelatine-only group was statistically similar to the IVM co-treatment groups, all of which had higher OCRs than the IVM-only group (Figures 5A,B).

Following treatment with oligomycin, an ATP synthase inhibitor, a negligible decrease in OCR was observed in the IVM-only group, indicating nearly zero ATP-linked respiration. Although post-oligomycin OCR levels were similar across all other groups, calculation of the OCR differences before and after oligomycin indicated comparable ATP-linked respiration among untreated, melatonin-only, and pinoline-only groups. In contrast, the agomelatine-only group exhibited an approximately sixfold reduction in ATP-linked respiration compared to the control. The IVM-treated groups co-treated with melatonin or agomelatine had substantially higher ATP-linked respiration, displaying 15- and 14-fold increases compared to the IVM-only group, respectively. The pinoline co-treatment group also showed a substantial increase in ATP-linked respiration compared to the IVM-only group, though to a lesser extent (Figures 5A,C).

After FCCP treatment, which uncouples mitochondrial oxidative phosphorylation to allow maximal respiration, the untreated, melatonin-only, and pinoline-only groups exhibited similar maximal respiration and reserve capacity (defined as the difference between basal and maximal OCRs). The agomelatine-only group displayed reduced maximal and reserve capacities at approximately 70% of the control. However, IVM groups co-treated with either melatonin or agomelatine had markedly improved maximal and reserve capacities compared to the IVM-only group, restoring these parameters to over than half the levels observed in the control. The IVM group co-treated with pinoline also showed a marked restoration of both maximal and reserve capacities, although the extent of recovery was less than that observed with melatonin or agomelatine cotreatment (Figures 5A,D,E).

### 3.6 Melatonin and agomelatine inhibit IVM-induced apoptosis in GC-1 spg

**FIGURE 6:**
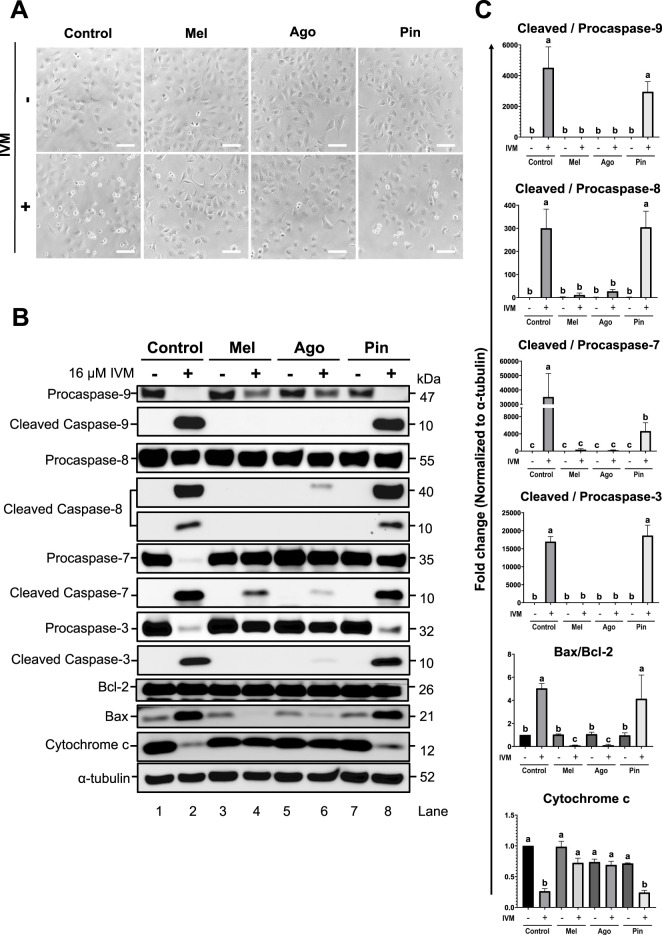
Attenuation of apoptosis by melatonin and agomelatine in GC-1 spermatogonia (spg) treated with ivermectin (IVM) for 3 h **(A)** Brightfield microscopy images showing morphological changes in GC-1 spg following treatment with IVM and/or melatonin analogs. Scale bars: 100 µm. **(B)** Western blot analysis of apoptosis-related proteins, including cleaved caspases, BCL-2, BAX, Cytochrome c, and α-tubulin (used as a loading control), under the indicated treatment conditions. Mel, melatonin; Ago, agomelatine; Pin, pinoline **(C)** Quantification of apoptosis-related proteins (n = 3). Data are shown as means ± SEM. Significant differences are denoted by different letters **(a–c)** at *p* < 0.05. Mel, melatonin; Ago, agomelatine; Pin, pinoline.

To evaluate the ability of melatonin and its analogs to inhibit apoptosis induced by 16 μM IVM, both morphological changes and expression levels of apoptosis-related proteins were assessed. Among the IVM-treated groups, a higher number of cells in the IVM-only and IVM + pinoline-cotreated groups exhibited apoptotic morphology compared to those co-treated with melatonin or agomelatine (Figure 6A).

A similar pattern was observed in the levels of activated caspases: the IVM-only and IVM + pinoline groups showed greater caspase activation than did the melatonin or agomelatine co-treated groups (Figure 6B). Melatonin and agomelatine inhibited caspase cleavage to different extents. Melatonin more effectively inhibited the cleavage of Caspase-8 and -3, whereas agomelatine more strongly inhibited Caspase-9 and -7. In the IVM-only and IVM + pinoline groups, no decrease in BCL-2 and an increase in BAX were observed. Both melatonin and agomelatine prevented these changes and even reduced BAX expression below the level of the control. The BCL-2 and BAX levels were unaffected by the treatment with melatonin or agomelatine alone. Consistent with previous findings (Chavez Varias et al., 2024), 16 μM IVM substantially decreased Cytochrome c levels. This reduction was also observed in the IVM and pinoline co-treatment group but not in cells co-treated with melatonin or agomelatine (Figures 6B,C).

## 4 Discussion

There is clear evidence linking IVM toxicity to redox imbalance, amplified by dysregulated ER–mitochondria crosstalk (Ahmed et al., 2020; Chahrazed et al., 2021; Chavez Varias et al., 2024; Cordeiro et al., 2024; Karaboduk et al., 2025). In the current study, we identified melatonin as an effective protector against mitochondrial injury in male germ cells exposed to IVM. Melatonin is an attractive cytoprotectant in this context due to its extremely low toxicity profile, with no established lethal dose. To investigate the influence of molecular structure on melatonin’s protective capacity, we also evaluated its two related analogs, agomelatine and pinoline. Our findings demonstrate that both melatonin and agomelatine exhibit mitochondria-specific cytoprotective effects. Agomelatine’s comparable ability suggests that melatonin analogs containing both a methoxy group and a *N*-acetyl side chain may inhibit mitochondrial toxicity. Melatonin’s role and biotechnological applications in preserving male fertility and reproduction have gained increasing interest (Sun et al., 2020; Assidi, 2022). Previous *in vitro* studies in GC-1 spg have shown that melatonin upregulates cell proliferation pathways in spermatogonia (Li et al., 2017; Zhu et al., 2018; Xu et al., 2023). Consistent with these findings, our results show increased translocation of Ki67 in melatonin-treated spermatogonia. In contrast, agomelatine did not induce this effect, suggesting that the indole-amine structure unique to melatonin plays a key role in promoting germ cell proliferation. However, under cytotoxic conditions, the focus shifts from increasing proliferation to preservation. In this context, both melatonin and agomelatine−but not pinoline−were effective in preventing proliferation loss and maintaining cell morphology and health.

Given the known mechanism of IVM-induced toxicity in male germ cells, we evaluated the efficacy of melatonin and its analogs in mitigating redox stress caused by ROS accumulation. Although all three molecules are reported to have antioxidant properties (De La Fuente Revenga et al., 2015; Rebai et al., 2021). Their effectiveness under toxic conditions was previously unclear. Our data showed that all three compounds reduced intracellular ROS, but melatonin and agomelatine produced the most pronounced effects. Furthermore, intracellular Ca^2+^ accumulation, which is associated with mitochondrial dysfunction and oxidative stress (Raimondi et al., 2021; Chavez Varias et al., 2024), was also substantially inhibited by melatonin and agomelatine, but not by pinoline. These findings align with improved cell proliferation and survival, highlighting the importance of ROS and Ca^2+^ homeostasis in the protective effects of these molecules.

Consistent with improvement in ROS and Ca^2+^ levels, IVM-induced losses in ΔΨm, mitochondrial mass, and overall mitochondrial respiration were inhibited by melatonin and agomelatine. However, caution has been raised regarding the use of MitoTracker for quantifying mitochondrial mass, as its fluorescence intensity may reflect oxidative stress rather than actual mass (Doherty and Perl, 2017). Despite this concern, our results showed an inverse relationship between extreme intracellular ROS accumulation and MitoTracker fluorescence, supporting its validity in this context. These direct observations of key mitochondrial parameters strengthen the connection between redox homeostasis and mitochondrial health. Additionally, the restoration of Cytochrome c levels−reduced by IVM−by melatonin and agomelatine may further explain how these molecules preserve mitochondrial function (Suofu et al., 2017). Thus, mitochondria represent a promising target for alleviating IVM-induced cytotoxicity.

Our findings also contribute to the understanding of melatonin’s chemical structure and its biological function. The indole-containing tryptamine core is believed to be primarily responsible for melatonin’s antioxidant and neuroprotective actions, while the 5-methoxy group enhances this effect (Letra-Vilela et al., 2016). Although agomelatine lacks the tryptamine core, its *N*-acetyl side chain and methoxy group may be sufficient to function as a mitochondria-directed cytoprotectant. However, agomelatine alone may reduce overall mitochondrial respiration in male germ cells. This supports the hypothesis that the tryptamine core is critical to melatonin’s action on mitochondrial complex IV. An *in vitro* study investigating mitochondrial toxicity of antidepressants reported that agomelatine can inhibit complex IV activity at high concentrations (10–100 μM) in isolated brain mitochondria (Ľupták et al., 2022). Choosing an ideal concentration of the drug is crucial, since agomelatine was previously reported to cause mild to moderate hepatotoxicity in repeat doses greater than or equal to the standard human dose (Gao et al., 2023). To address this, we used a ten-fold lower concentration and applied it uniformly across all melatonin analogs tested. At this dose, agomelatine did not disrupt the mitochondrial mass, ΔΨm, and redox homeostasis, nor did it induce cell death or loss of proliferation. Moreover, it restored mitochondrial respiration under toxicological contexts.

This study provides important new insights into the cellular mechanisms underlying IVM-induced germ cell toxicity, albeit limited by its *in vitro* design using GC-1 spermatogonia and short-term exposure conditions that may not fully reflect the complex physiology of the testes. Nevertheless, the findings of this study provide merit to assessing the cytoprotective properties of melatonin and agomelatine in refined models of spermatogenesis, such as 3D and/or organ culture, to materialize their translational relevance in agricultural, veterinary, and human health contexts as cytoprotectants. This work also offers an early yet valuable foundation for further *in vivo* studies and long-term exposure models, which will be essential for translating these results into real-world reproductive health applications. Given the widespread use of IVM in both agricultural and clinical settings, our results raise important concerns about its potential impact on male fertility. The observed protective effects of melatonin and agomelatine in preserving spermatogonial cell integrity under toxic stress warrant follow-up studies on their potential as adjunctive or precursor therapeutic agents for safeguarding male reproductive health from redox stress.

## 5 Conclusion

Melatonin exerts protective effects on the male reproductive system, largely through its antioxidant and mitochondria-supporting actions. In this study, we show that melatonin and agomelatine exhibit mitochondria-associated cytoprotective activity against IVM-induced male germ cell toxicity, in contrast to structurally distinct analog pinoline. Our findings further suggest that specific structural features of melatonin analogs may influence their capacity to restore redox homeostasis during toxic stress. These findings provide important preliminary insights for the future design of mitochondria-targeted therapeutics aimed at preserving male fertility and reproductive health.

## Data Availability

The raw data supporting the conclusions of this article will be made available by the authors, without undue reservation.

## Abbreviations

DMEM, Dulbecco’s modified Eagle’s medium, EDTA, ethylenediaminetetraacetic acid, ER, endoplasmic reticulum, ETC, electron transport chain, FBS, fetal bovine serum, IVM, ivermectin, mPTP, mitochondrial permeability transition pore, OCR, oxygen consumption rate, ROS, reactive oxygen species, spg, spermatogonia, SRB, sulforhodamine B, TBEA, Trypan Blue exclusion assay.
